# Compatibility of personality and major among freshman undergraduate nursing students of the Kerman University of Medical Sciences

**Published:** 2010

**Authors:** Abbas Abbaszadeh, Fariba Borhani, Mohaddeseh Mohsenpour

**Affiliations:** * Associate Professor, Department of Medical Surgical Nursing, School of Nursing and Midwifery, Kerman University of Medical Sciences, Kerman, Iran; ** Department of Medical Surgical Nursing, School of Nursing and Midwifery, Kerman University of Medical Sciences, Kerman, Iran

**Keywords:** Personality, nursing students, professional role

## Abstract

**BACKGROUND:**

Personality traits are major effective factors on student’s learning, educational achievements and employer’s job satisfaction. Metacognitive characteristics such as personality are only changeable up to 30% in the best educational condition. Therefore, students should be evaluated for such characteristics including their personality compatibility with their major. The present study investigated the personality compatibility of freshman undergraduate nursing students of the Kerman University of Medical Sciences in 2008 with nursing profession.

**METHODS:**

This was a descriptive study using a standard questionnaire based on Holland’s career and personality theory on 82 freshman nursing students of Kerman University of Medical Sciences in 2008. Data were analyzed using SPSS software.

**RESULTS:**

More than 50% of the participants evaluated their information of nursing profession average. The personality of 41.3% was not compatible with nursing profession and the personality of 26.2% was relatively compatible. Only 32.5% of the participants had completely compatible personalities with this profession.

**CONCLUSIONS:**

Considering the limitations of the present study and previous studies, further studies are recommended. It seems that students’ knowledge of majors and careers are increasing, but it is necessary to plan and make more effort to recognize personal characteristics and personality compatibility with professions. Knowledge of professions and personalities along with each other are valuable and neglecting one would be an obstacle to achieve goals including decreasing job resignation, increasing job efficiency and satisfaction.

Personality traits are major effective factors on learning approaches^1^, educational achievements^2^,^3^ and employer’s job satisfaction.^4^ Students’ image of their clinical ability is very significant and based on their real abilities and personality type.^5^ Efficiency and clinical judgment of students are affected by many factors including IQ, psychological characteristics, personality, supports, teachers and stress. Personality traits such as neurotic and extrovert can put students at risk of stress.^6^ Job successes can happen when the individual characteristics as well as their career features are evaluated. Based on this double recognition and understanding the relationship between these two, the appropriate job can be chosen correctly.^2^ In difficult jobs such as nursing, along with physical abilities, psychological characteristics should be considered as important factors of career.^4^ Metacognitive characteristics such as personality are only changeable to 30% in the best educational conditions.^7^,^8^ Therefore, people should be evaluated based on these characteristics when they enter a profession to assure their personality compatibility with the job.^7^ There are many studies on personality type of nursing students such as Karami,^3^ Nikpour et al,^9^ Hejazi and Irani^10^ in Iran, Warbah et al^6^ in India and Zhang^11^ in China and the effects of personality on nurses’ efficiency and clinical judgment such as Pulford and Suha^5^, Chamorro-Premuzic and Reichenbacher^12^ in England, Kaufman et al in Spain,^13^ Saka and Gati^14^ in Israel and Fabio and Busoni^15^ in Italy; but on the personality compatibility with nursing profession just one study by Adib and Dianati was carried out which more than half students of Kashan and Tehran did not have compatible personalities with nursing profession.^16^ The result of this study showed the incompatibility of more than 50% of students with this profession, while this study just considered the first dominant personality. However, if three dominant personalities were considered, the results might have shown a clearer picture of nursing students’ compatibility with their profession.

It seems that a study on nursing students’ personality compatibility with their profession can help better understanding of the subject, considering the lack of studies on this topic. This study cannot find the answer to all questions related to students’ personality compatibility and nursing profession, but such studies can provide a background for further studies to investigate the subject more precisely. Studies on this subject can provide a background for planning methods in selecting students and criteria to use the personality compatibility as a factor in accepting students. Therefore, this study is conducted hoping that it can enter the personality compatibility as a criterion for accepting nursing students in order to improve the students’ progress in cognitive skills and especially clinical skills. Considering the strong effect of personality in performance, ability, educational achievements and job satisfaction of students and nurses and considering that there is no individual characteristics selection before entering a profession in Iran, this study investigates the personality compatibility of freshman nursing students of Kerman University of Medical Sciences in 2008 with this profession. The results of this study may help planning for students’ selection in nursing profession, career and management as well as providing a background for further studies.

## Methods

In this descriptive study, sampling was census and the sample size was the same as the study population (82 students). All freshman students of nursing in Kerman University of Medical Sciences in day time shift who entered on October 2008, including freshman nursing students of Kerman, Bam and Zarand, who were willing to participate in the study, were invited.

A Persian translation of the standard questionnaire based on Holland’s theory^17^ which was used in a study by Adib and Dianati in 2001 was used for data collection to assess personality compatibility. Reliability of this questionnaire is assessed so many times and r has been always between 0.8 and 0.9.^2^ This questionnaire includes five sections on career expectations, activities, competencies, jobs and selfassessments in six factor typology of social, artistic, investigative, conventional, enterprising and realistic. Each typology has some scores. Considering the students’ answers, they take a total score for each part and the three highest scores show their dominant personality. The higher scores have no significance in personality and just the rank from high to low show the dominant personality. Following the questionnaire guidelines, typologies compatible with nursing profession was defined for each person in three levels of compatible, relatively compatible and incompatible. At the beginning of the questionnaire, after mentioning ethical considerations and asking a question of demographic data, the level of students’ information about nursing from their viewpoint was assessed. Then, the researcher introduced the study and explained ethical considerations such as secrecy of data and permission to leave the study at any time they want. Then the questionnaires were distributed and collected after completion. Collected data were analyzed with SPSS software. To compare the personality compatibility percentage in different groups of demographic data, the chi-square test was used and to determine the personality compatibility percentage, frequency distribution tables were used.

## Results

A total of 82 students participate in this study. Two questionnaires were not useful because of lack of data in personality compatibility section. The mean age of participants was 18.92 with standard deviation of 1.63. Demographic data of participants are presented in table 1. Eight students (9.8%) defined their level of information on nursing major high, 54 students (66.7%) defined it average, 17 students (21%) defined it low and 2 students (2.5%) said that they had no information about this major. Personality compatibility was determined considering the 3 dominant typology and based on the questionnaire guidelines. Those whose 3 first typology (the highest scores of each part) was social, investigator and artistic considered to be completely compatible. Those whose 2 higher scores were social and investigator, regardless of their third typology were considered relatively compatible and the rest were considered incompatible. The highest scores of each person was the dominant typology (Table 2). In total, considering the 3 highest scores for each student, 41.3% of students were not compatible with nursing profession and 26.2% had relative compatibility and just 32.5% were completely compatible with this major. The personality compatibility in the two genders was significantly different (p < 0.001) and 48% of female students were compatible with the profession while this percentage was 6.7% for male students. Moreover, there were significant differences between various groups based on different levels of parents’ education, marital status, caring or not caring to know the results (p < 0.05). The difference of students’ level of information about nursing was not significant in any of the demographic variables. Also, there was no significant difference in students’ personality compatibility with various levels of information.

**Table 1:** Participants’ personality compatibility in terms of demographic data

	Personality Compatibility									
Variable	Compatible		Relatively compatible		Incompatible		Total		*χ*^2^	*P*
	Number	Percentage	Number	Percentage	Number	Percentage	Number	Percentage		
Sex									15.01	> 0.001
Male	2	6.7	12	40	16	53.3	30	37.5		
Female	24	48	9	18	17	34	50	62.5		
Residence									0.54	> 0.05
Local	20	33.3	14	23.3	26	43.4	60	75.9		
Non-local	6	31.6	6	31.6	7	36.8	19	24.1		
Father’s education									11.95	^*^0.015
Less than high school	9	22	15	36.6	17	41.4	41	62.6		
High school	13	56.5	1	4.3	9	39.2	23	29.5		
University degree	4	28.6	6	28.6	6	42.8	14	17.9		
Mother’s education									11.81	^*^0.013
Less than high school	10	22.2	18	40	17	37.8	45	57		
High school	13	52	2	8	10	40	25	31.6		
University degree	3	3.33	1	11.1	5	55.6	9	11.4		
Marital status									5.82	^*^0.028
Single	26	34.7	21	28	28	37.3	75	93.7		
Married	0	0	0	0	5	100	5	6.3		
School									4.47	> 0.05
Bam	10	40	6	24	9	36	25	30.5		
Zarand	5	3.26	8	42.1	6	31	19	25.6		
Kerman	11	30.6	7	19.4	18	50	36	43.9		
Work experience									3.48	> 0.05
Have	0	0	1	20	4	80	5	6.5		
Do not have	26	31.1	19	26.4	27	37.5	72	93.5		
Mean score of high school									3.16	> 0.05
Over 17	18	40.9	10	22.7	16	36.4	36	45		
Less than 17	8	22.2	11	30.6	17	47.2	44	55		
Frequency of taking Konkoor									5.02	> 0.05
One time	19	33.9	15	26.8	22	39.3	56	70		
Two times	7	38.9	5	27.8	6	33.3	18	22.5		
Three times	0	0	1	33.3	2	66.7	3	3.75		
Four Times	0	0	0	0	3	100	3	3.75		
Information level									5.83	> 0.05
High	3	37.5	1	12.5	4	50	8	10		
Average	19	35.8	16	30.2	18	34	53	67		
Low	4	25	4	25	8	50	16	20		
No information	0	0	0	0	2	100	2	3		

Some participants have not answered to all questions; therefore, the total of all numbers are not the same. The difference was significant.

**Table 2:** Frequency and percent distribution of the first dominant typology in freshman nursing students in Kerman University of Medical Sciences in 2008

First dominant personality	Social	Conventional	Artistic	Investigative	Enterprising	Realistic	Total
Frequency	46	1	5	22	2	4	80
Percentage	57.5	1.3	6.2	27.5	2.5	5	100

## Discussion

In general, the present study showed the students’ favorable understanding of nursing major from their own viewpoints, so that 71% evaluated their information of the major higher than average. However, only 32.5% had personalities compatible with this profession. This can explain the lack of knowledge about personal interests and characteristics or wrong information about choosing majors or importance of marginal factors in choosing career. Holland is his theory of career and personality emphasizes that compatibility of career environment and personality brings more success, job satisfaction and productive work life.^18^ Considering that nursing profession needs characteristics such as patience, restraint, sincerity, social manner, cooperation, loving human beings, etc. in addition to professional knowledge, having a personality compatible with the major helps the health of the society. In studies of Brodie et al^19^ and Buerhause et al in the U.S^20^ and Vanhanen and Jahnhonen in Finland^21^, students’ perception of nursing profession changed during their education and there was a significant difference between the first year and the 4^th^ year of the study. In the study of Abdi in Iran in 2004, the mean of students’ information acquisition was 48% in different majors^22^ and in the present study it was 71% based on students’ self-assessment. In the study of Adib and Dianati, 11.5% of participants mentioned sufficient information, 40.4% mentioned an image near medicine, 17.3% mentioned lack of information and 30.8% mentioned absence of information^16^ while in the present study, absence of information was just mentioned by 2.5% that means relatively improvement in students’ information about their major. This difference can be related to the difference questions asked from students in these studies or can be related to the real improvement of students’ information due to the recent educational programs such as educational guidance of high school students, adding the obligatory course of higher education plan to know various careers, activity of mass media in choosing majors and students’ more attention to selecting majors and knowing various educational majors.

In the only found study that used Holland questionnaire of students’ personality compatibility with nursing profession, 44.2% of participants were not compatible with this profession.^16^ The results of the present study (41.3%) agrees with those result, but considering the difference in the method of analyzing the questionnaire in that study, there are lots of differences. In the study of Adib and Dianati in Kashan and Tehran, personality compatibility was assessed just with one typology (the highest scores or the first dominant typology) but according to Holland’s theory, at least 3 first typologies (or the three highest scores) are required for personality compatibility with the profession.^23^ Also, the return questionnaires was 96.7% in the present study, while it was 68.1% in Adib and Dianati study.^16^ The main limitation of this study was the length of questionnaire which may have effected participants answers. But they were given a short break to fix this limitation. In addition, a safe one way connection was given to those students who wanted to know the results of their questionnaire to give them more motivation for seriously answering. The results of the questionnaire of students were sent to them at the end of analysis. Considering these limitations, a correct comparison cannot be done between the results of these two studies and they cannot explain a logical procedure to improve or worsen the students’ personality compatibility with nursing profession. Further studies seem useful on this topic.

There was a significant difference in personality compatibility of female and male students that can be due to higher sensitivity of female students in choosing their majors. It is possible that male students consider other factors such as economic factors rather than personality compatibility in choosing professions. In other cases, since the size of the groups was not matched, comparison is not reliable. For example, just 5 of the participants were married and all of them had incompatible personalities with the profession. Further studies are needs in this case as well.

In general, considering the increase of students information about nursing profession and considering that this increase have not change the personality compatibility of students with the major, we think that programs to introduce university majors and their corresponding careers to students in Iran have been significantly increases, even though not completely well-developed. However, students need to know themselves and their interests and personalities and their compatibility with the major they choose. Studying in a major that is compatible with personality depends on the knowledge of that major and knowing one’s interests and personality and how one is compatible with that major. These two together and along with each other are valuable and paying attention to one without the other cannot achieve the goals that include decrease in leaving jobs, increase in efficiency and job satisfaction etc. As it is seems, these two are not sufficient in students who enter university.

The Authors declare that have no conflict of interest in this study. The ethical committee approved the study.
